# The kinetics of chirality assignment in catalytic single-walled carbon nanotube growth and the routes towards selective growth†
†Electronic supplementary information (ESI) available: Details of density functional theory (DFT) calculations, definition of interfacial formation energy (IFE), cap formation energy and fitting equation, Fig. S1–S4 and Table S1. See DOI: 10.1039/c7sc04714b


**DOI:** 10.1039/c7sc04714b

**Published:** 2018-02-19

**Authors:** Ziwei Xu, Lu Qiu, Feng Ding

**Affiliations:** a Centre for Multidimensional Carbon Materials , Institute for Basic Science , Ulsan 44919 , Korea; b School of Materials Science and Engineering , Ulsan National Institute of Science and Technology , Ulsan 44919 , Korea . Email: f.ding@unist.ac.kr; c Institute of Textiles and Clothing , Hong Kong Polytechnic University , Hong Kong S.A.R. , China; d School of Materials Science & Engineering , Jiangsu University , Zhenjiang 212013 , China

## Abstract

Depending on its specific structure, or so-called chirality, a single-walled carbon nanotube (SWCNT) can be either a conductor or a semiconductor. This feature ensures great potential for building ∼1 nm sized electronics if chirality-selected SWCNTs could be achieved. However, due to the limited understanding of the growth mechanism of SWCNTs, reliable methods for chirality-selected SWCNTs are still pending. Here we present a theoretical model on the chirality assignment and control of SWCNTs during the catalytic growth. This study reveals that the chirality of a SWCNT is determined by the kinetic incorporation of pentagons, especially the last (6^th^) one, during the nucleation stage. Our analysis showed that the chirality of a SWCNT is randomly assigned on a liquid or liquid-like catalyst surface, and two routes of synthesizing chirality-selected SWCNTs, which are verified by recent experimental achievements, are demonstrated. They are (i) by using high melting point crystalline catalysts, such as Ta, W, Re, Os, or their alloys, and (ii) by frequently changing the chirality of SWCNTs during their growth. This study paves the way for achieving chirality-selective SWCNT growth for high performance SWCNT based electronics.

## Abstract 2

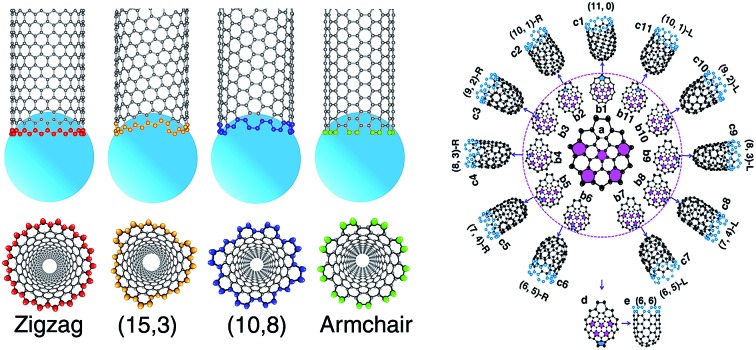
The routes towards carbon nanotube's chirality control during growth was revealed by kinetic modelling.

## 1. Introduction

Due to their numerous potential applications, great efforts, including chirality-selective growth and post-growth selection, have been dedicated for a reliable method of producing SWCNTs with desired chiralities. Although significant advances have been achieved in post-growth selection, such as the selection of more than 10 different SWCNTs of high purity by DNA wrapping^1^ or gel chromatography,^2^ it suffers from the drawback of only selecting short SWCNTs with low yields and low quality due to chemical functionalization and high expenses. Therefore the direct growth of chirality-selected SWCNTs is the most desired method for achieving this goal.

Over the last two decades, significant attempts have been made towards the growth of chirality-selected SWCNTs.^3^–^7^ But the achievements in the early years were very limited.^3^–^7^ Before 2014, the best technique was only able to grow a single type of SWCNT (*e.g.*, (6,5)^5^,^6^ and (9,8)^8^) with ∼50% selectivity, which is far from that required for high performance electronics, with >99.9% selectivity. Such limited progress of direct growth was attributed to the lack of understanding of the SWCNT growth mechanism, especially the mechanism of chirality assignment and control during SWCNT nucleation and growth.

In the past, great efforts, such as various molecular dynamics and Monte Carlo simulations, were dedicated to understanding the chirality of SWCNTs and to seeking potential means of its control.^9^–^21^ These studies have revealed many details of SWCNT cap nucleation and indicated that the growth of SWCNTs is determined by both thermodynamic and kinetic processes^12^,^14^,^22^–^26^ but how the chirality of a SWCNT was assigned during the growth remains a mystery.

Here we present a theoretical analysis of SWCNT nucleation. The current study demonstrates that the nucleation of a SWCNT is a kinetic process, and its chirality is randomly assigned if it is grown on a liquid catalyst particle. Based on such insightful understanding, the mystery that hindered the chirality-selective SWCNT growth is revealed, and two potential routes towards the chirality-selective SWCNT growth were proposed.

## 2. Results and discussion

**Fig. 1:**
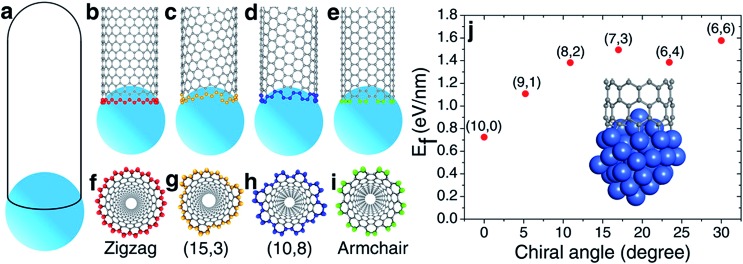
The formation of the single-walled carbon nanotube (SWCNT)–catalyst interface. (a) The vapor–liquid–solid (VLS) model of SWCNT growth. (b–e) Atomic details of (18,0), (15,3), (10,8), and (9,9) SWCNTs on the liquid catalyst particles. (f–i) Bottom view of the tube end. (j) The interfacial formation energies (IFEs) of a series of SWCNTs on the liquid-like Ni_55_ catalyst particle.

According to the vapor–liquid–solid (VLS) growth mechanism, the scenery of catalytic SWCNT growth is that an open end of a SWCNT is attached to a spherical liquid catalyst particle (Fig. 1(a)). In such a continuous model, the differences among SWCNTs of different chiralities are ignored. However, at the atomic level, the open end of a SWCNT is a carbon ring of armchair (AC), zigzag (ZZ), or chiral (Fig. 1(b–i)), through which the chirality of a SWCNT can be recognized by the catalyst.^27^

It was widely argued that a specific type of SWCNT can be nucleated in a larger number if it has a more stable interface with the catalyst.^7^,^19^,^20^ The validity of such a mechanism for chirality-selective growth is expected because each type of SWCNT has a unique open end attached to the catalyst surface (Fig. 1(b–e)). Fig. 1(j) shows the interfacial formation energies (IFEs) of a series of SWCNTs (details of IFE and its calculation are shown in the ESI†) with very similar diameters, on the liquid Ni_55_ catalyst particle, calculated by the density functional theory (DFT) method incorporated in the VASP (Vienna *Ab initio* Simulation Package).^28^,^29^ The generalized gradient approximation (GGA) with the Perdew–Burke–Ernzerhof (PBE) function was employed during the calculations.^30^ As expected, a systematic variation of the IFE, that the small chiral angle SWCNTs have lower IFE, can be clearly seen. This indicates that a small chiral angle SWCNT is more stable. If the nucleation process of a SWCNT is near thermal equilibrium, the number of each type of SWCNT can be estimated by

1 *N* ∼ Exp(–*E*_f_/*k*_b_*T*),

where *E*_f_ is the IFE of the SWCNT on the catalyst surface, *k*_b_ is the Boltzmann constant, and *T* is the experimental temperature of SWCNT growth, which is mostly in the range of 1000–1300 K. As shown in Fig. 1(j), the difference in IFE varies in the range of 0.7–1.6 eV nm^–1^. For a typical SWCNT of diameter *d* ∼ 1 nm, the maximum IFE difference is Δ*E*_f_ ∼ 0.9 eV nm^–1^ × (π × *d*) ∼ 3.0 eV. Thus, the maximum population difference among these SWCNTs reaches exp(–Δ*E*_f_/*k*_b_*T*) ∼ exp(–30) ∼ 10^–14^. The high ratio between the populations of different types of SWCNTs clearly indicates great potential for achieving chirality-selection in SWCNT growth. For example, the (10,0) zigzag SWCNT shows exceptional stability and its population in the sample can be estimated to be greater than 99%.

Despite the above analysis of the potential for achieving chirality-selective SWCNT growth, it has never been observed in experiments. In contrast, the as-grown SWCNT samples produced by experiments normally contain SWCNTs of various chiralities from AC to ZZ and normally have no huge differences among their populations.^27^,^31^,^32^

Most previous experiments showed an even chiral angle distribution of SWCNTs, which drastically contradicts the quasi-thermal-equilibrium analysis based on eqn (1).^7^ Such a puzzle can only be solved by one of the two routes: (i) there is actually no systematic IFE difference among SWCNTs of different chiral angles, or (ii) the nucleation of SWCNTs cannot be described by eqn (1), or the assignment of the chirality of SWCNTs is a process that is far from thermal equilibrium. The DFT calculations shown in Fig. 1(j) and in previous studies^7^,^20^ confirmed that the IFE depends on the edge structure or the chirality of the SWCNT. Therefore, the only route to solve the contradiction is that the assignment of the chirality of SWCNTs is a kinetic process which is far from thermal equilibrium. It is worth noting that this conclusion is in agreement with previous studies.^12^,^14^,^24^

To achieve a deep insight into the kinetics of the chirality assignment of SWCNTs in the catalytic growth, we recall the scenery regarding the birth of a SWCNT. The nucleation of a SWCNT starts from the aggregation of carbon atoms to form a small sp^2^ carbon network on a catalyst particle surface.^33^,^34^ Then, guided by the curved catalyst particle surface, the network grows into a graphitic cap by adsorbing carbon atoms to form polygons, such as hexagons and pentagons. Without considering other defects besides the pentagons (*e.g.*, heptagons, octagons, sp^3^ or dangling atoms), once the number of the pentagons in the graphitic cap reaches six, the cap becomes a mature hemisphere and can be considered as a SWCNT infant because its chirality is uniquely determined by the relative positions of the six pentagons. Based on the addition of C_2_ radicals to the AC sites of the rim of the cap of a SWCNT, Balbuena *et al.* proved that the growth of the caps of armchair or near-armchair SWCNTs is favoured both thermodynamically and kinetically.^35^ In the following growth process, the addition of more carbon atoms into the SWCNT infant leads to the elongation of the SWCNT, without altering the chirality because of the efficient topological defect healing during growth.^27^,^36^

**Fig. 2:**
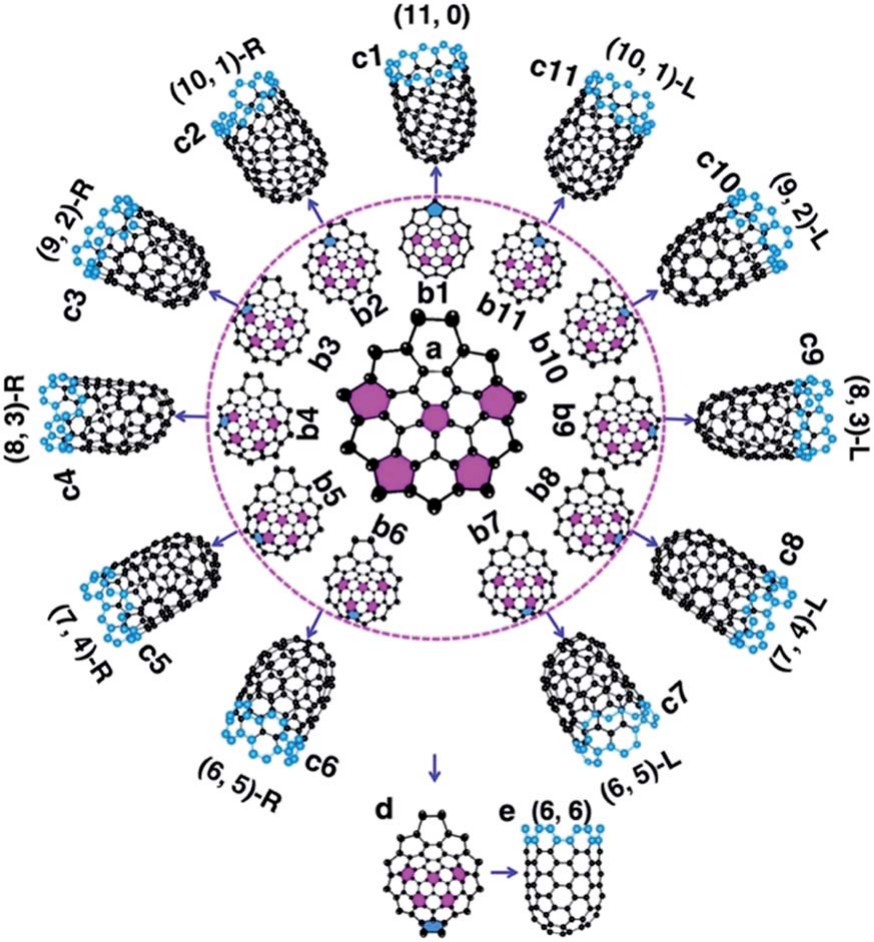
The nucleation of a SWCNT from a graphitic cap with five pentagons. (a) An immature graphitic cap with 5 pentagons. (b1–b11) The eleven options of adding the 6^th^ pentagon into the cap when a new polygon ring is formed, and the resultant SWCNTs (c1–c11). (*n*,*m*)-L and (*n*,*m*)-R denote the left handed and right handed chiral SWCNTs, respectively. (d → e) The formation of the (6,6) SWCNT.

To understand how the arrangement of the six pentagons in the cap determines the chirality of a SWCNT, a cap with five pentagons is considered [Fig. 2(a) and more examples are shown in Fig. S4†]. For such a cap, the incorporation of one more pentagon is required to turn it into a SWCNT with well-defined chirality. In the cap shown in Fig. 2(a), there are eleven options of incorporating the 6^th^ pentagon during the addition of a new polygon ring (Fig. 2(b1–11)), which turn the cap into eleven different SWCNTs, which are (11,0) and right handed and left handed (10,1), (9,2), (8,3), (7,4) and (6,5) SWCNTs, respectively (Fig. 2(c1–11)). These possibilities cover all the population of the (*m*,*n*) SWCNTs of the *n* + *m* = 11 family. If the last (6^th^) pentagon was formed during the addition of the next polygon ring, we can have all the possible caps of the *n* + *m* = 12 family SWCNTs, such as the (6,6) armchair SWCNT, as shown in Fig. 2(d → e). Similarly, adding the 6^th^ pentagon far away from the cap central leads to the formation of a large SWCNT with any arbitrary chiral angle. This analysis clearly indicates that (i) the position of the 6^th^ pentagon fully controls the chirality of the SWCNT regardless of the locations of the other five pentagons in the cap and (ii) SWCNTs with different chiral angles have the same probabilities of being nucleated if the addition of the 6^th^ pentagon to the rim of the cap is random. The above finding simplifies the origin of the chirality of a SWCNT during nucleation, because only the 6^th^ or the last added pentagon matters.

It's important to note that although the first five pentagons are formed in the central area of the cap and their relative positions are hardly changed during further enlargement of the cap, the above analysis can be applied to the enlargement of any cap (see Fig. S4† for another example). Therefore, the 1^st^ to 5^th^ pentagons having fixed locations is not a precondition of the conclusion.

Based on the above analysis, we can conclude that a SWCNT of any chiral angle has the same probability of being nucleated, if the addition of the 6^th^ pentagon is not site-selective. At the atomic level, as can be seen in Fig. 2(a) → (b2–11), the addition of the 6^th^ pentagon in most sites occurs in a very similar manner: adding two carbon atoms to a ZZ site on the rim of the cap (except Fig. 2(a) → (b1)). For the VLS SWCNT growth, a liquid catalyst droplet has an isotropic surface, which ensures an identical environment around each site of the cap edge. Thus, in the VLS SWCNT growth, the probability of adding a pentagon onto any location of a cap edge is expected to be equal.

During crystal growth, it is known that the formation of particles smaller than the nucleus is a process of quasi–thermal equilibrium and the population of each particle can be estimated using eqn (1) because of the reversibility of the formation process. While the formation of particles greater than the size of the nucleus is a kinetic process that is normally far from thermal equilibrium because of the irreversibility of atomic addition during the following crystal growth process. For example, the crystal shape during growth can be determined by applying the kinetic Wulff construction but not the Wulff construction.^37^

**Fig. 3:**
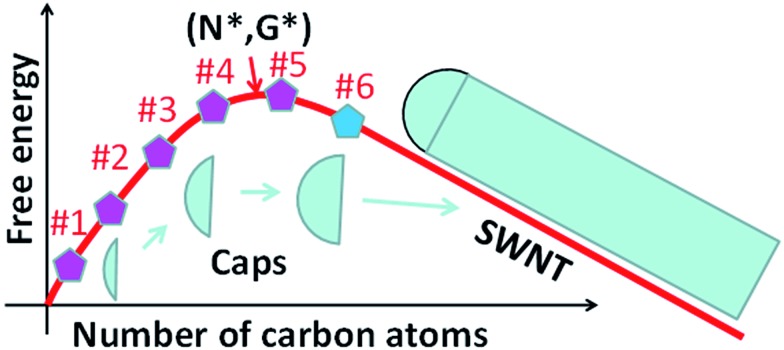
The Gibbs free energy evolution during the nucleation and the growth of SWCNTs. The N* and G* denote the size of the nucleus and the nucleation barrier of the SWCNT. After the addition of the 6^th^ pentagon, *N* > *N*_6_, the cap becomes matured and the further elongation of the SWCNT thereafter results in a linear drop of the Gibbs free energy.

Whether the size of a mature cap with 6 pentagons exceeds the size of the nucleus is critical for the validation of the above analysis. To answer this question, let's consider the evolution of the Gibbs free energy, *G*, during the growth of a SWCNT. As illustrated in Fig. 3, the nucleation of a SWCNT starts with a small graphitic cap. Initially, the addition of C atoms into the cap raises the cap formation energy sharply because of its large circumference to area ratio. Then the energy rise becomes slower and slower as the edge to volume ratio becomes smaller and smaller. At the size of the nucleus, N*, *G* reaches the maximum, G*. As discussed above, once the 6^th^ pentagon is formed in the cap, the cap becomes a SWCNT infant with a unique chirality. Thus, beyond the formation of the 6^th^ pentagon, further addition of carbon atoms leads to the elongation of the SWCNT wall in a repeatable manner and a linear decrease of *G*. So, the first derivative of the *G* curve, at *N*_6_, where the 6^th^ pentagon was added, must be negative or equal to zero. Therefore, we can conclude that the cap size at which the 6^th^ pentagon was added must be greater than the critical size of the nucleus, or *N*_6_ > N* (Fig. 3). Because the formation of the 6^th^ pentagon leads to an energy drop, the reverse process, the elimination of the 6^th^ pentagon, is a process involving an energy rise. So, different from the pentagon formation before the nucleation (*i.e.*, *N* < N*), which is reversible and thus the population of each can be estimated using eqn (1), the formation of the 6^th^ pentagon or the assignment of the chirality during the growth of a SWCNT is an irreversible kinetic process that cannot be described by eqn (1).

**Fig. 4:**
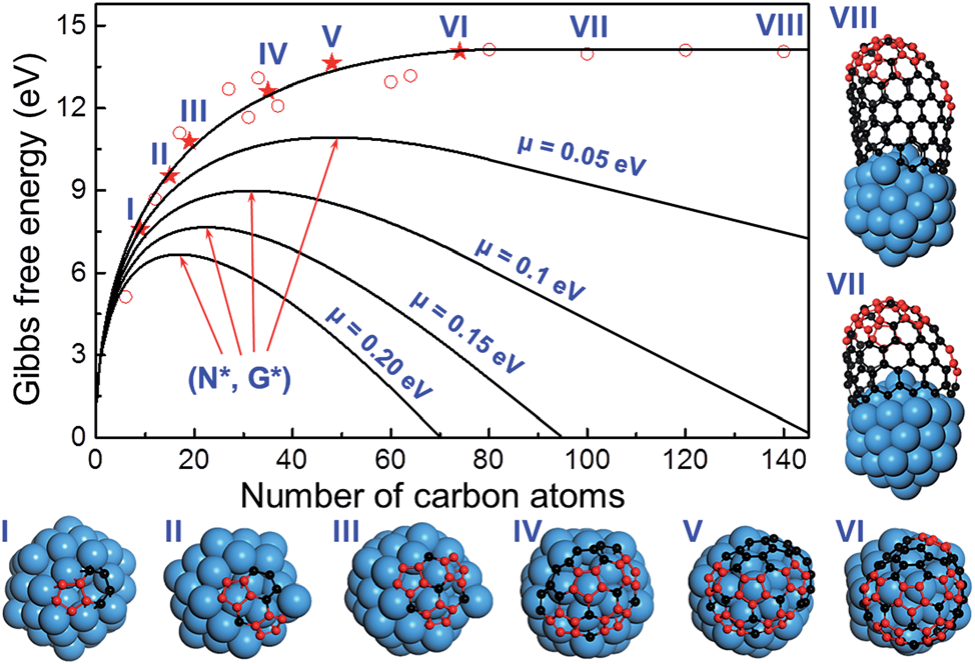
The formation energies of graphitic caps of different sizes and short (*n*,*m*) SWCNTs of different lengths. The caps with different numbers of pentagons (from one to six) and short SWCNTs with a mature cap (that has six pentagons) are shown. It can be clearly seen that the tube part of the energy curve is perfectly linear and the first derivatives of both parts are continuous. Therefore, we can conclude that, under the conditions of SWCNT growth, or if the chemical potential difference between a carbon atom in feedstock and in SWCNTs, *μ*, is greater than zero, the maximum of the Gibbs free energy curve or the nucleation point must appear before the location of the sixth pentagon.

To further verify the kinetic addition of the 6^th^ pentagon, we calculated the nucleation process of a (11,1) SWCNT on a Ni_55_ catalyst surface and the results are shown in Fig. 4. From the calculation, we can clearly see that the formation energy evolution of the cap can be well fitted with a smooth curve and the elongation of the SWCNT accompanies a linear variation of the formation energy (see the fitting equation in the ESI†). Besides, the linear part and the curve can be well connected at the location of the 6^th^ pentagon with their first derivatives being exactly the same. Under the conditions of SWCNT growth, the Gibbs free energy is given as follows:

2 *G* = *E*_f_ – *N* × *μ*,

where *E*_f_ is the formation energy of the CNT cap (see the definition in the ESI†) and *μ* is the chemical potential difference between a carbon atom in feedstock and in CNTs. After the nucleation point, *G* keep going down with the increasing of *N*, or the first derivative of the *G* ∼ *N* curve at *N*6 is a negative value (Fig. 4). Consequently, we can see that the maximum of the Gibbs free energy or the nucleation point was shifted to N* = 52, 34, 23, and 18 for *μ* = 0.05, 0.1, 0.15 and 0.2 eV, respectively. All these critical sizes are smaller than the size of the cap with six pentagons, *N* = 78. This calculation verifies the analysis shown in Fig. 3 and confirms that the addition of the last pentagon to the rim of the graphitic cap during SWCNT growth is beyond the cap nucleation size and can be considered as a kinetic process.

**Fig. 5:**
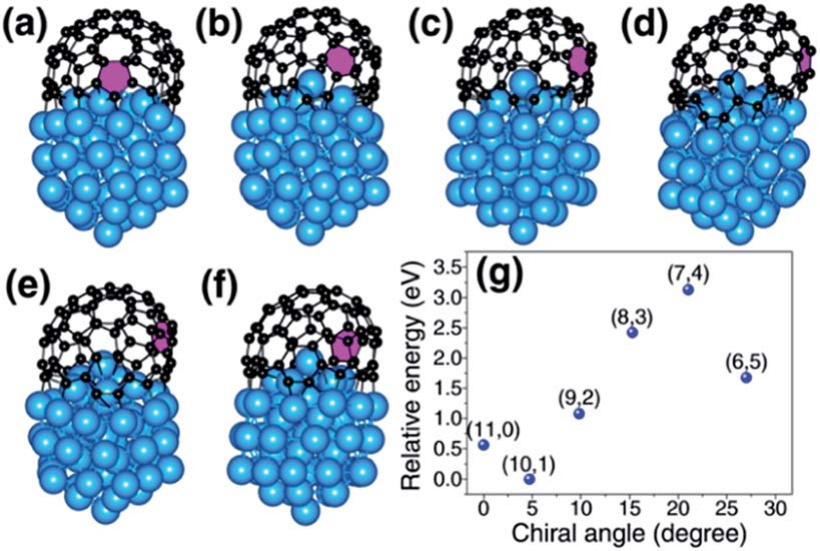
The relative energy of various matured graphitic caps (with six pentagons) on an icosahedral Ni_55_ solid catalyst particle. (a–f) The caps which correspond to SWCNTs of the *n* + *m* = 11 family, where the only difference of these caps is the location of the last pentagon (marked in purple). (g) The relative energies of the caps *vs.* the chiral angles of the corresponding SWCNTs.

The above analysis indicates that the addition of the 6^th^ pentagon during the SWCNT nucleation is a kinetic process, with equal probability at every potential site of the cap in the VLS growth. This successfully explains the broad experimental observations that there is no chiral angle selection in most VLS SWCNT samples, although the IFEs between the SWCNTs and catalysts are very different. It is important to note that an isotropic surface of the liquid catalyst particle is assumed in drawing such a conclusion. Experimental observations have shown that SWCNT or multi-walled carbon nanotube (MWCNT) growth on a solid catalyst particle *via* the vapor–solid–solid (VSS) mechanism is also possible.^38^–^40^ On a solid crystalline catalyst surface, there is a possibility that the sites along the circumference of a graphitic cap can be differentiated by the local environment of the anisotropic catalyst surface.^41^,^42^ In such a circumstance, the formation of the 6^th^ pentagon could be site-selective and, thus, chirality-selective nucleation of SWCNTs or MWCNTs could be achieved. As shown in Fig. 5, on an icosahedral Ni_55_ surface, the addition of the 6^th^ pentagon along the circumference of the cap leads to a systematic change of the formation energy of up to 2.0 eV. Among these sites, the two sites that correspond to the (10,1) and (6,5) SWCNTs show exceptional stability near the ZZ and AC edges, respectively.

Experimentally, the (6,5) SWCNTs have been synthesized in very large abundance in the low temperature (∼600–800 °C) CVD growth process by using Co or CoMo as the catalyst (CoMoCat),^5^,^6^ in agreement with the above theoretical analysis. At such a low temperature, these catalysts may retain their solid crystalline structures. We also noticed that the chirality selection abruptly disappears at 850 °C or higher temperature.^6^ Such a transition can be well explained by reasonably assuming that the melting point of a CoMo alloy particle with a diameter of ∼1–2 nm supported by the substrate is between 800 and 850 °C.^43^ Similarly, the preferential growth of conducting SWCNTs was also attributed to the catalyst formation with sharp edges, or, in other words, the catalyst must have a crystalline structure.^3^

The high-quality growth normally requires high temperature (>900 °C),^44^ one of the critical variables (others like appropriate carbon/metal and metal/substrate interactions) of the CVD experiment which affects the quality of the grown SWCNTs. But the most frequently used catalysts (Fe, Co and Ni) do not have high enough melting points to maintain the crystalline structure of the nanoparticles of only a few nanometres. For this reason, there is normally no chirality selection in most SWCNT samples grown at high temperature. By simply examining the periodic table, the suitable candidates of metal catalysts could be tantalum, tungsten, rhenium, osmium (the four metal elements whose melting points are about 3000 °C) or their alloys.

With this understanding, we propose the design of high melting point catalysts to maintain the crystallinity of the catalyst particle as the route towards the chirality-selective growth of SWCNTs. Following such a route, in recent years, through elaborate design of alloy catalysts (*e.g.*, W_6_Co_7_, MoC_2_, and WC), SWCNTs with a few different types of chiralities (*e.g.*, (12,6), (16,0), (14,4), and (8,4)) are synthesized with purity as high as 90%.^45^–^48^

**Fig. 6:**
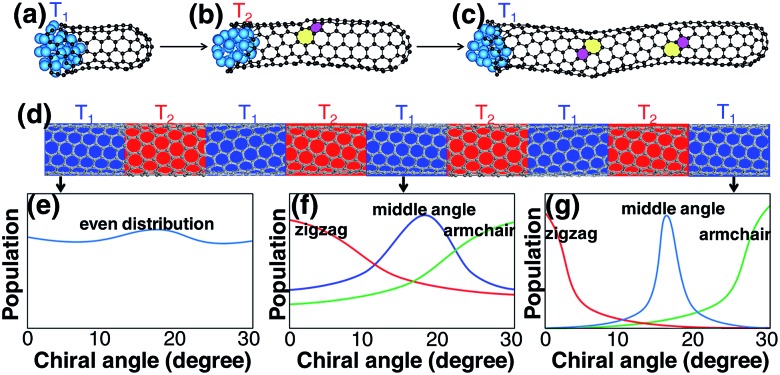
(a → c) The slight change of the temperature during the SWCNT elongation can lead to the change of the SWCNT chirality. Repeated change of the growth temperature (d) can drive the even chiral-angle distribution of initial SWCNTs to the final narrowed distribution with the most stable interfacial formation energy (e → g). The three colors in (f) and (g) represent three narrowed chiral-angle distributions of near zigzag, middle chiral angle and near armchair, respectively.

Although a SWCNT tends to maintain its original chirality originating from the cap-to-SWCNT transition by the efficient healing of the topological defects,^44^ varying the growth conditions may alter its chirality. It was proved that varying the growth temperature slightly during its growth could change the chirality of a SWCNT.^49^ For the change of the chirality of a SWCNT during SWCNT elongation, the IFE must play an important role. Based on this understanding, we propose another route to achieve chirality-selection in growth by changing the growth conditions periodically, such as temperature, to drive the SWCNT to the types with the most stable interfaces with the catalyst particles (Fig. 6). Based on this strategy, a new CVD method, named “tandem plate CVD”, has been realized with the near-zigzag chirality enriched (∼72%) *via* varying the temperature of growth periodically.^50^

## 3. Summary and conclusions

In summary, through DFT calculations, a systematic change of the interfacial formation energy between SWCNTs and catalysts was identified. Thus, the experimental puzzle of no chiral angle selection during SWCNT growth was successfully explained by the kinetic addition of 6^th^ pentagon into a graphitic cap during SWCNT growth. The previous experimental observations of the abundance of a few types of SWCNTs synthesized at low temperature were also reasonably explained by using the solid catalyst particle assumption. Based on this understanding, two strategies of achieving chirality-selective growth in VLS and VSS growth were proposed: (i) by using high melting point catalysts (Ta, W, Re, and Os) or their alloys as catalysts, and (ii) by changing the chirality of SWCNTs during the elongation. We believe that the present analysis on the chirality of SWCNTs will motivate more and more experimental studies and eventually lead to the success of chirality-selective SWCNT growth and applications in carbon based electronics.

## Conflicts of interest

There are no conflicts to declare.

## Supplementary Material

Supplementary information
