# Supplementary material for: The kinetics of chirality assignment in catalytic single-walled carbon nanotube growth and the routes towards selective growth
Source: Chem Sci. 2018 Feb 19;9(11):3056–61. doi: 10.1039/c7sc04714b (PMC5916013; doi:10.1039/c7sc04714b)
Supplement: Supplementary file 1 [file SC-009-C7SC04714B-s001.pdf]

Electronic Supplementary Material (ESI) for Chemical Science.
This journal is © The Royal Society of Chemistry 2018

Supplementary Information of

The Kinetics of Chirality Assignment in Catalytic Single-Walled Carbon Nanotube Growth and the Routes Towards Selective Growth

Ziwei Xu,[c,d] Lu Qiu and Feng Ding[a,b,c*]

[a] *Centre for Multidimensional Carbon Materials, Institute for Basic Science, Ulsan 44919, S. Korea*

[b] *School of Materials Science and Engineering, Ulsan National Institute of Science and Technology, Ulsan 44919, S. Korea*

[c] *Institute of Textiles and Clothing, Hong Kong Polytechnic University, Hong Kong S.A.R., China;*

[d] *School of Materials Science & Engineering, Jiangsu University, Zhenjiang 212013, The People's Republic of China*

*** E-mail:** f.ding@unist.ac.kr

S-1: Details of density functional theory (DFT) calculation

DFT calculations are performed with the VASP (Vienna Ab-initio Simulation Package) [1,2]. The generalized gradient approximation (GGA) is adopted for the exchange correlation by using Perdew-Burke-Ernzerhof (PBE) functional, with the spin polarization taken into account [3]. The plan wave cutoff energy is set to be 400 eV and the projector-augmented wave (PAW) is used as the pseudopotential [4]. The convergence criterion for energy and force is set to be 10^{-4} eV and 0.01 eV/Å, respectively.

S-2: Definition of interfacial formation energy (IFE)

The IFE (E_f) of a SWCNTs on a liquid Ni_{55} is

$$E_f = E_{FE} - E_b \qquad \text{(S1)}$$

in which E_{FE} and E_b are the formation energy of the free SWCNT end and the SWCNT-metal binding energy, respectively, and E_b is

$$E_b = E_{NT} + E_{Ni} - E_{NT@Ni} \qquad \text{(S2)}$$

in which $E_{NT@Ni}$ is the energy of SWCNT attached on Ni_{55}, E_{NT} and E_{Ni} are energies of the isolated SWCNT and Ni_{55}, respectively. E_{FE} in Eq. (S1) is

$$E_{FE} = 0.5*(2*E_{NT2} - E_{NT1}) \qquad \text{(S3)}$$

in which E_{NT1} is the energy of a longer SWCNT and E_{NT2} is the energy of a shorter SWCNT, which is obtained by cutting the longer SWCNT into two equal segments (see Fig. S1). The factor 0.5 refers the fact that two open ends are formed when a SWCNT is cut into two SWCNTs (see Fig. S1).

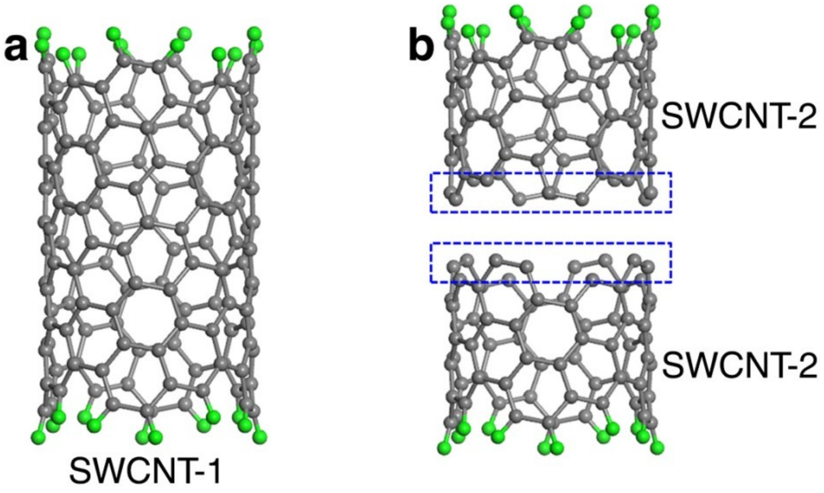

Fig. S1 Schematic diagram of (a) SWCNT-1 and (b) SWCNT-2 for the calculation of the formation energy E_{FE} of the free SWCNT end. The green atoms are hydrogen atoms. Two open ends (blue dashed rectangular) are formed by cutting the longer SWCNT-1 into two shorter SWCNT-2 segments. The E_{FE} is defined as Eq. (S3).

S-3. Liquid Ni_{55} particle formation

To obtain a liquid Ni_{55} particle, MD simulation with Sutton-Chen potential [5] is performed at 1,500 K, which is greatly above the melting point of the cluster, for 10 ns. Randomly selected liquid Ni_{55} structures in the MD simulation were used to represent the liquid catalyst particles in the IFE calculation (see Fig. S2).

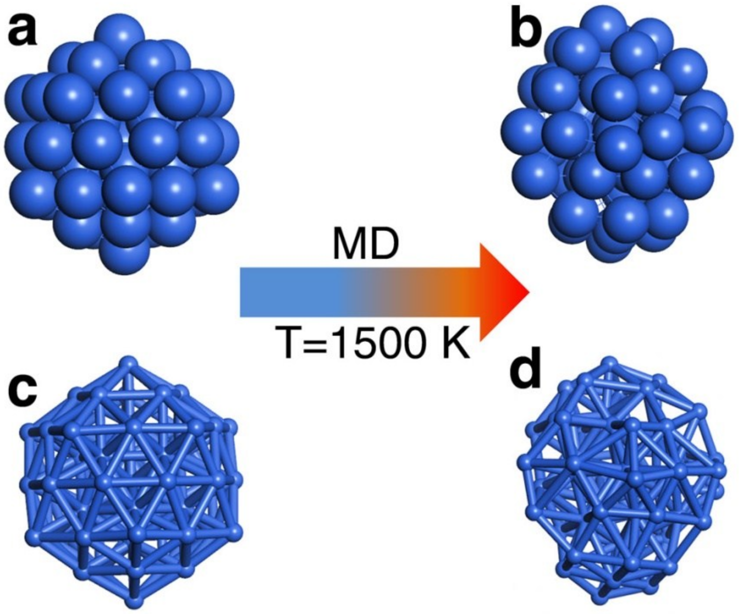

Fig. S2 An icosahedral cluster of Ni_{55} is melted at 1,500 K and liquid particles are obtained during the MD simulation. (a→b) CPK model and (c→d) ball-stick model of the solid and liquid Ni_{55} particles.

S-4: The IFE calculation

To achieve a reasonable IFE for each SWCNT attached to the liquid Ni_{55} catalyst particle, six different melting Ni_{55} structures are used for DFT calculations and thus six IFEs are obtained for each SWCNT as shown in Fig. S3. Among the six IFEs, the smallest one is used to represent the IFE of a SWCNT-catalyst interface as plotted in Fig. 1(j) of the main text considering the high probability of it being formed during SWCNT growth. It's worth to note that the statistics of the data and the calculated IFEs of SWCNTs attached a solid icosahedral catalyst particle represent same trend (see Fig. S3).

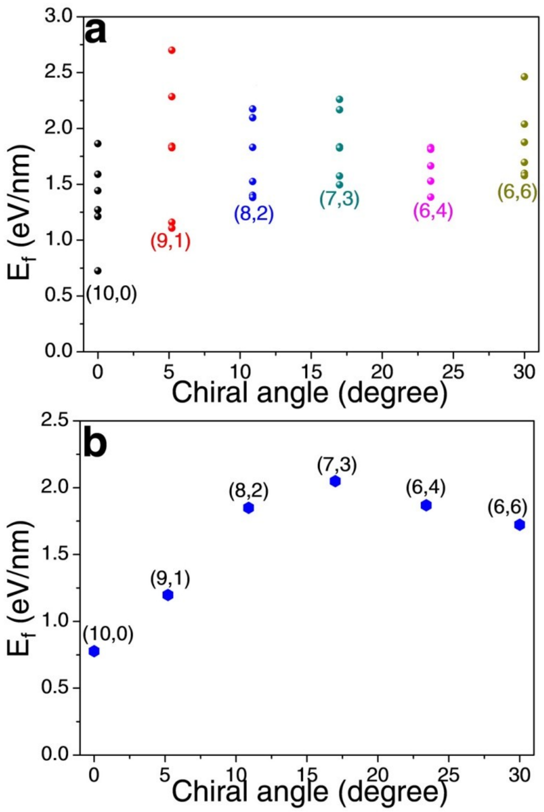

Fig. S3 IFEs calculated by attaching SWCNTs onto liquid and solid Ni_{55} particles. (a) The six interfacial formation energies (IFEs) calculated by attaching each SWCNT onto six different liquid catalyst particles with the DFT method (see the methods). The detailed values are listed in table S1. (b) IFEs calculated by attaching SWCNTs onto solid catalyst particle (icosahedral Ni_{55} cluster).

Table S1. The interfacial energies (IFEs in eV/nm) calculated by attaching SWCNT onto liquid Ni_{55} particles with the DFT method. #1 - #6 refers six liquid Ni_{55} particles. The lowest IFE for each SWCNT is marked in bold.

IFE / **CNT**	#1	#2	#3	#4	#5	#6
(10,0)	1.8637	1.5896	1.2123	1.2719	1.4431	**0.7245**
(9,1)	1.8258	2.2845	**1.1074**	1.1591	2.7004	1.8410
(8,2)	1.8294	1.4007	1.5244	**1.3816**	2.1737	2.0951
(7,3)	**1.4950**	1.5729	2.2597	2.1671	1.8351	1.8242
(6,4)	1.828	1.8118	1.5276	**1.3847**	1.6636	1.8160
(6,6)	1.6959	2.4621	2.0387	**1.5764**	1.8761	1.5971

S-5: The random chirality assignment from other caps with five pentagons.

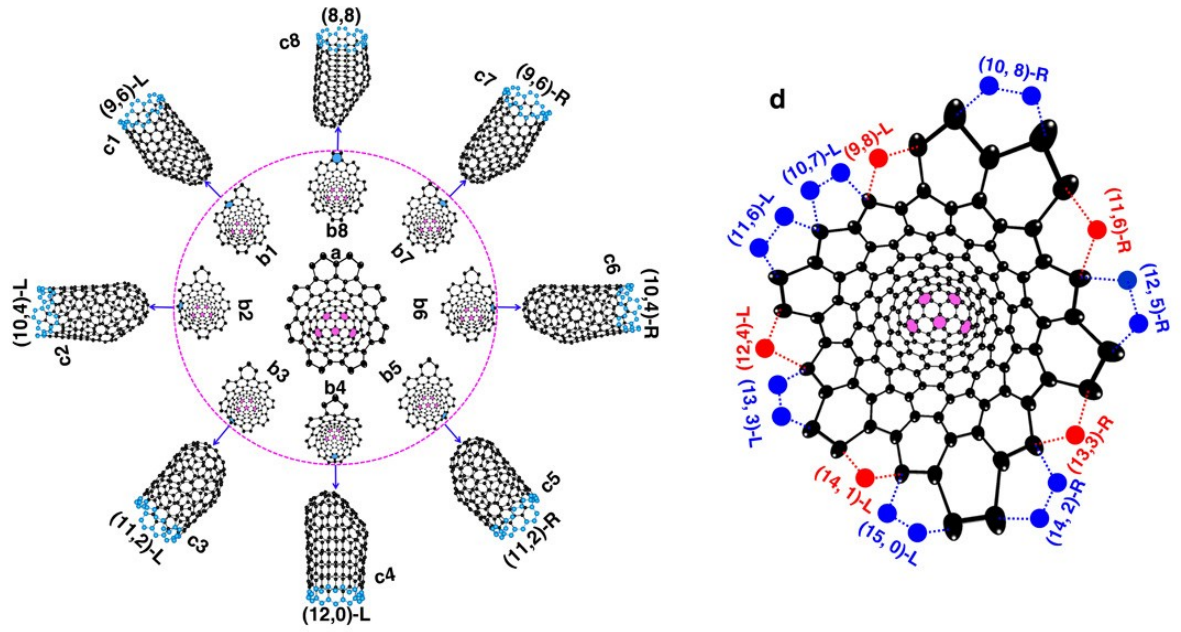

Fig. S4 (a) An graphitic cap with seven AC and one ZZ sites on the edge. (b1-b8) the eight options of forming the 6th pentagon into the cap and the resulted SWCNTs (c1-c8). (n,m)-L and (n,m)-R denote the left handed and right handed chiral SWCNTs, respectively. (d) The chiral indexes of the resulted SWCNTs by adding the 6th pentagon onto different edge sites of the cap which is mixed with AC and ZZ sites. It can be clearly seen that the resulted SWCNTs have randomly assigned chiralities either the cap edge is dominated by AC sites or mixed with AC and ZZ sites.

S-6: Definition of formation energy cap and the fitting equation

The formation energy of the CNT cap is calculated as the following formula

$$E_f = E_{CNT@Ni} - E_{Ni} - N * \varepsilon_C, \quad (1)$$

where $E_{CNT@Ni}$ is the total energy of the CNT on the Ni_{55}, E_{Ni} is the energy for the isolated Ni_{55} particle, N is the number of the carbon atoms of CNT and ε_C is the energy of per carbon atom in CNT.

The data of E_f as function of N can be fitted by the equation below with standard errors lower than 0.05 for all the parameters:

$$E_f = \frac{1}{0.01558 + 0.30038 \times N^{-\frac{1}{2}}} - 0.07689 \times N \quad (2)$$

The fundamental form of the equation 2 is based on the nucleation of the SWCNT cap and the elongation of the SWCNT stem. The formation energy therefore includes energetic contributions from the edge atoms and the inner atoms, respectively. During the growth of the 2D cap, when the atom's number N is small, the formation energy of the edge atoms (E_{edge}) is proportional to the

perimeter (l) of the 2D cap, namely $E_{edge} \sim l \sim N^{-1/2}$. However, when the N is large enough to form a SWCNT, l becomes the perimeter of the SWCNT and the E_{edge} approaches to a constant. Combining these two considerations, we define the equation of $E_{edge} = 1/(a+b^* N^{-1/2})$, which implies $E_{edge} \sim N^{-1/2}$ for small N and $E_{edge} \sim 1/a$ for large N. For the inner atoms, their increase will bring forth a linear decrease of the energy, namely $E_{inner} \sim N$. So, the total formation energy can be fitted by using $E_f = E_{edge} + E_{inner} = 1/(a+b^* N^{-1/2}) - c^*N$. The exact parameters of a, b, and c are fitted empirically according to the data in Fig. 4 calculated by the DFT method

References

1. Blöchl, P. E. Projector augmented-wave method. *Phys. Rev. B* **50**, 17953-17979 (1994).
2. Kresse, G.&Furthmüller, J. Efficient iterative schemes for ab initio total-energy calculations using a plane-wave basis set. *Phys. Rev. B* **54**, 11169-11186 (1996).
3. Perdew, J. P., Burke, K.&Ernzerhof, M. Generalized gradient approximation made simple. *Phys. Rev. Lett.* **77**, 3865-3868 (1996).
4. Kresse, G.&Joubert, D. From ultrasoft pseudopotentials to the projector augmented-wave method. *Phys. Rev. B* **59**, 1758-1775 (1999).
5. Sutton, A. P.&Chen, J. Long-range finnis-sinclair potentials. *Philosophical Magazine Letters* **61**, 139-146 (1990).
